# Supplementary material for: Calcium transients regulate the apical emergence of basally located progenitors during Xenopus skin development
Source: Nat Commun. 2025 Jul 19;16:6650. doi: 10.1038/s41467-025-61610-7 (PMC12274442; doi:10.1038/s41467-025-61610-7)
Supplement: Supplementary file 1 — Supplementary Information [file 41467_2025_61610_MOESM1_ESM.pdf]

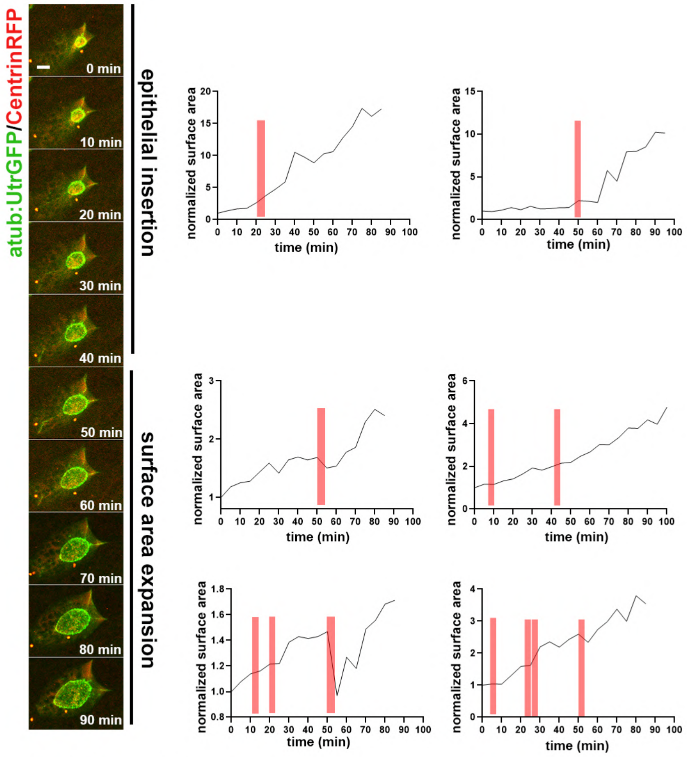

Supplementary Figure 1. Calcium transients precede epithelial insertion and apical cell surface area expansion.

Left: Stills from a time-lapse recording showing different phases of MCC apical emergence. Right: Quantification of apical surface area of single MCCs during Epithelial insertion and surface area expansion phases of apical emergence. Red bars represent Ca2+ transients. Ca2+ transients precede the increase of the apical surface area in all cells. Scale bar:5um

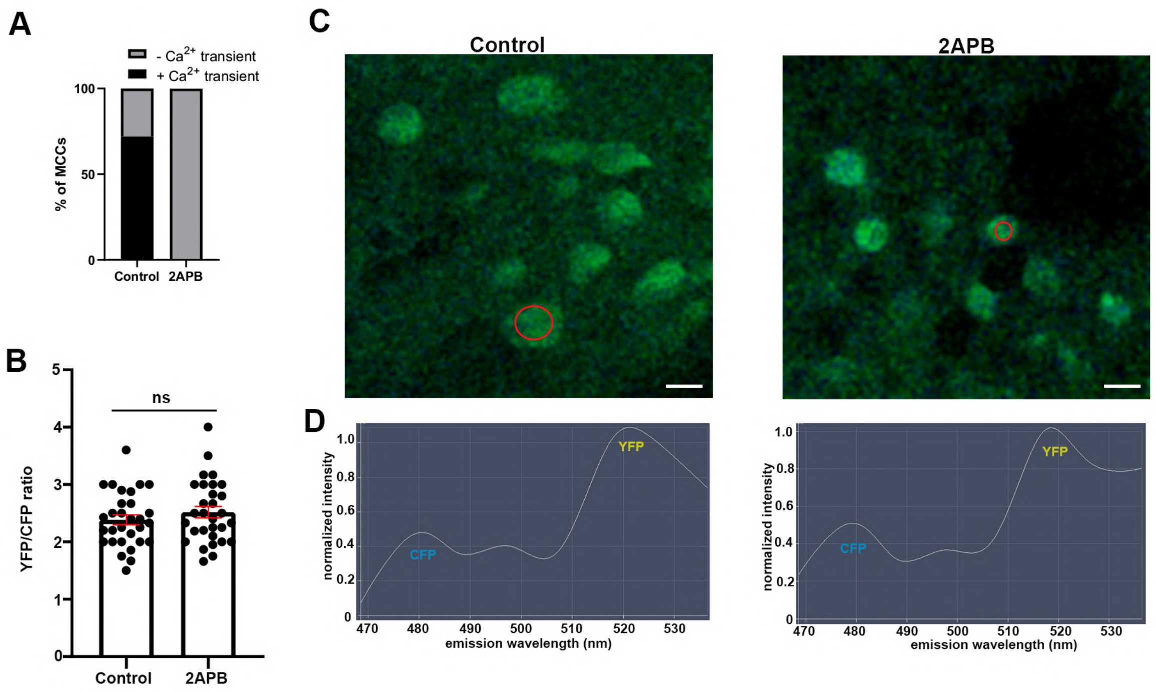

Supplementary Figure 2. 2APB treatment affects calcium transients but not basal calcium levels.
A) Quantification of Ca2+ transient events in MCCs from control and 2APB treated embryos. n=100 MCCs from 5 control embryos and 100MCCs from 5 2APB treated embryos. $\chi 2$ test ; ****p<0.0001. B) Quantification of YFP/CFP ration from Cameleon expressing MCCs from control and 2APB treated embryos. Two-sided unpaired student's t test; p= 0.318 ; mean ± SEM. n=30MCCs from 3 control and 3 2APB treated embryos') Representative spectral images of Cameleon expressing MCCs in control and 2APB-treated embryos. D) Cameleon emission spectrum in representative MCCs from C. Spectral imaging reveals the distinct emission peaks from CFP and YFP upon excitation with 458nm laser. Scale bars: 20um.

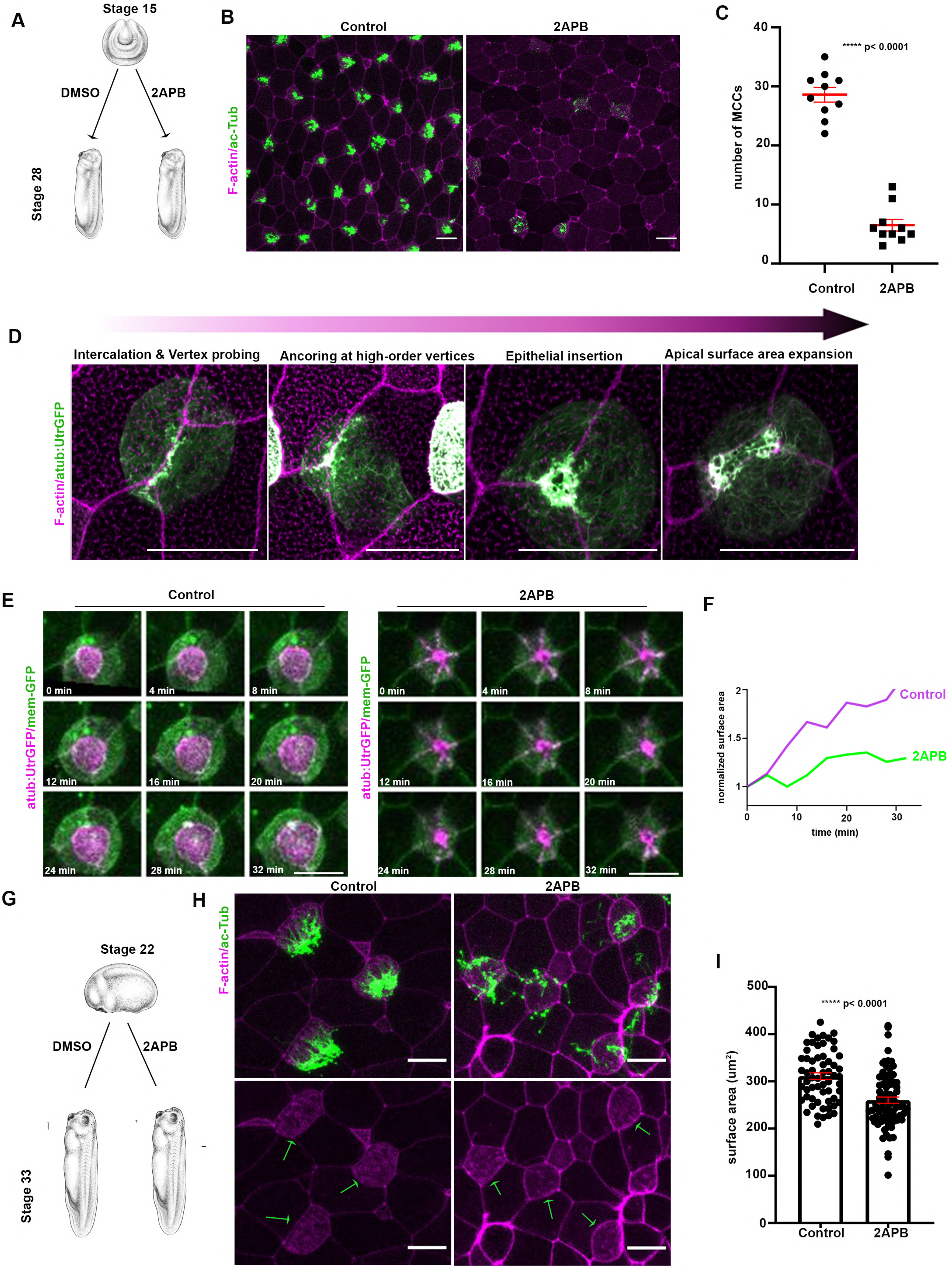
A
Stage 15
DMSO
2APB
Stage 28
B
Control
2APB
F-actin/ac-Tub
C
***** p< 0.0001
number of MCCs
40
30
20
10
0
Control
2APB
D
Intercalation & Vertex probing
Ancoring at high-order vertices
Epithelial insertion
Apical surface area expansion
F-actin/atub:UtrGFP
E
Control
2APB
atub:UtrGFP/mem-GFP
0 min
4 min
8 min
12 min
16 min
20 min
24 min
28 min
32 min
F
normalized surface area
time (min)
Control
2APB
G
Stage 22
DMSO
2APB
Stage 33
H
Control
2APB
F-actin/ac-Tub
I
***** p< 0.0001
surface area (um²)
500
400
300
200
100
0
Control
2APB

Supplementary Figure 3. Calcium transients are necessary for MCC apical emergence.
A) Schematic for the experimental protocol used to assess MCC apical emergence in B. B) Representative images of control and 2apb-treated skin epithelium of stage 28 embryos. MCCs have successfully been inserted into the skin epithelium of control embryos. MCCs apical emergence is defective in 2apb-treated embryos. C) Quantification of MCC epithelial insertion. Two-sided unpaired student's t test; ****$p < 0.0001$; mean ± SEM. n=10 positions from 5 control embryos and 10 positions from 5 2apb-treated embryos. D)Main steps of MCC apical emergence. E) Stills from time-lapse recordings of MCCs upon binding to high-order vertices. MCC in control embryo successfully inserts into the overlying skin epithelium, expanding its surface area. The MCC in the 2APB-treated embryo displays defective epithelial insertion after its binding to a high-order vertex. F) Quantification of cell surface area over time in control and 2apb-treated MCC from E. G) Schematic for the experimental protocol used to assess MCC apical emergence H. H) Representative images of control and 2APB treated skin epithelium of stage 33 embryos. I) Quantification of MCCs apical area from stage 33 control and 2APB treated embryos. Two-sided unpaired student's t test; ****$p < 0.0001$; mean ± SEM. n=70 MCCs from 5 control embryos and 65 MCCs from 5 2apb-treated embryos. Scale bars: 20um. Xenopus embryo illustrations, ©Natalya Zahn (2022)..

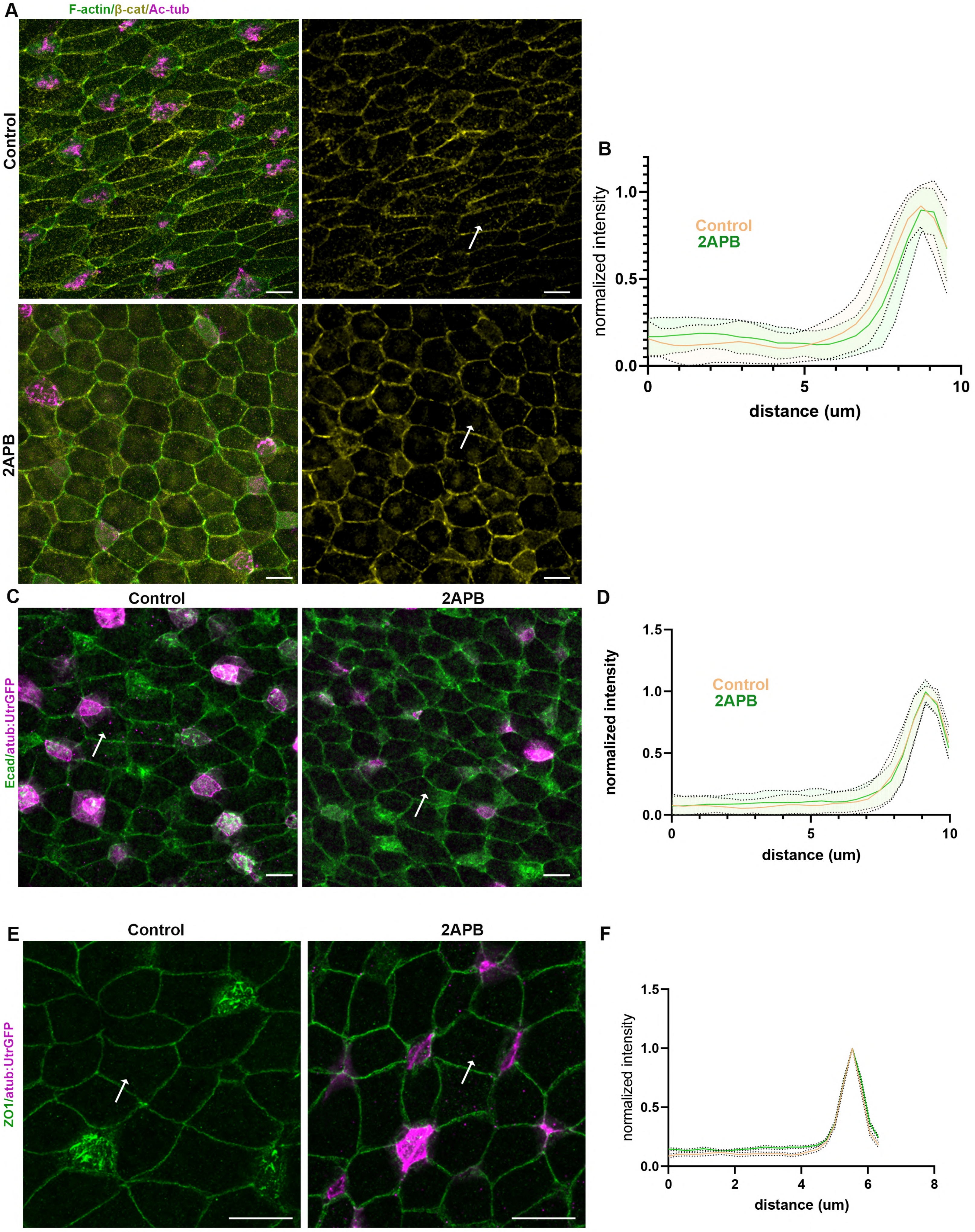

A
F-actin/β-cat/Ac-tub
Control
2APB
B
normalized intensity
1.0
0.5
0.0
Control
2APB
0
5
10
distance (um)
C
Control
2APB
Ecad/atub:UtrGFP
D
1.5
1.0
0.5
0.0
normalized intensity
Control
2APB
0
5
10
distance (um)
E
Control
2APB
ZO1/atub:UtrGFP
F
1.5
1.0
0.5
0.0
normalized intensity
0
2
4
6
8
distance (um)

Supplementary Figure 4. 2APB treatment does not affect epithelial integrity.
A) Representative images of the skin epithelium of control and 2apb-treated embryos stained with β-catenin. B) Quantification of β-catenin cortical enrichment. Multiple t-tests, $p>0.05$. n=23 cells from 3 control embryos and 30 cells from 3 2apb-treated embryos. C) Representative images of the skin epithelium of control and 2apb-treated embryos stained with E-cadherin. D) Quantification of E-cadherin cortical enrichment. Multiple t-tests, $p>0.05$. n=30 cells from 3 control embryos and 30 cells from 3 2APB treated embryos E) Representative images of the skin epithelium of control and 2APB treated embryos stained with ZO-1. F) Quantification of ZO-1 cortical enrichment. Multiple t-tests, $p>0.05$. n=30 cells from 3 control embryos and 30 cells from 3 2APB treated embryos. Scale bars: 20um.

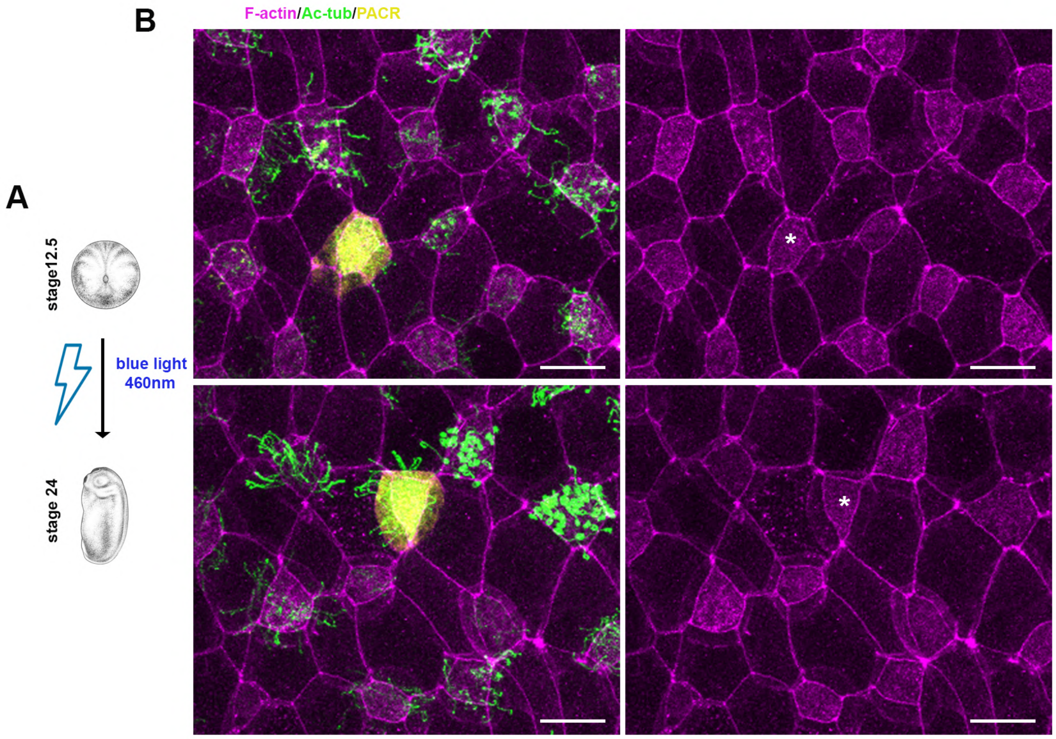

Supplementary Figure 5. Exposure to blue light rescues the PACR-induced defects.
A) Experimental procedure followed to assess the impact of PACR expression in the presence of blue light. B) Representative images of the skin epithelium from stage 25 embryos exposed to blue light. MCCs expressing PACR (asterisks) do not display defects in apical emergence. Scale bars: 20um. Xenopus embryo illustrations, ©Natalya Zahn (2022).

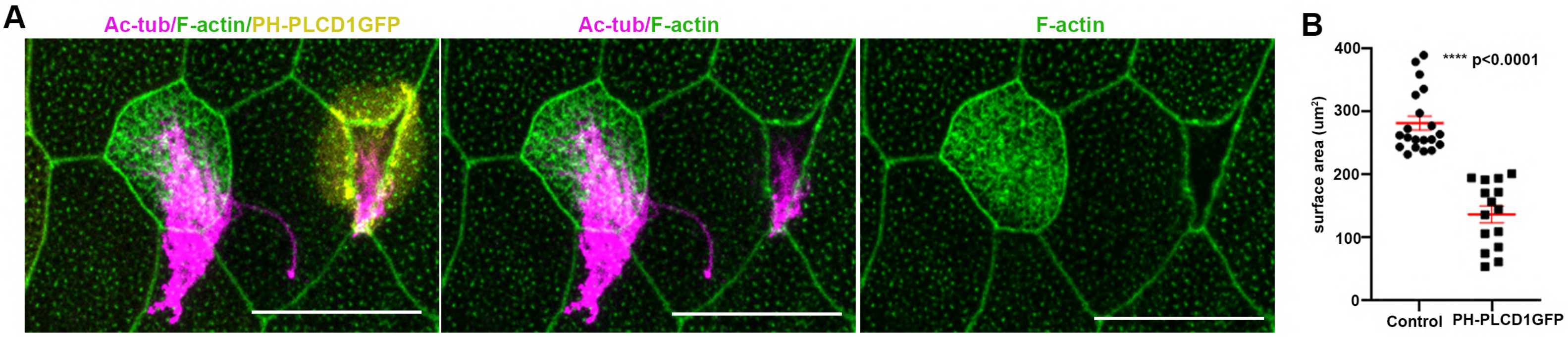

Supplementary Figure 6. Expression of a PLC dominant negative results in defective MCC apical emergence.

A) Representative image from stage 30 embryo. MCC expressing the PLC dominant negative PH-PLCD1GFP (yellow) displays defective apical emergence characterized by reduced apical surface area. B) Quantification of MCC apical surface of Control and PH-PLCD1GFP expressing MCCs of stage 30 embryos. Two-sided unpaired student's t test; ****$p < 0.0001$; mean ± SEM. n=20 control MCCs and 15 PH-PLCD1GFP expressing MCCs from 4 different embryos. Scale bars: 20um.

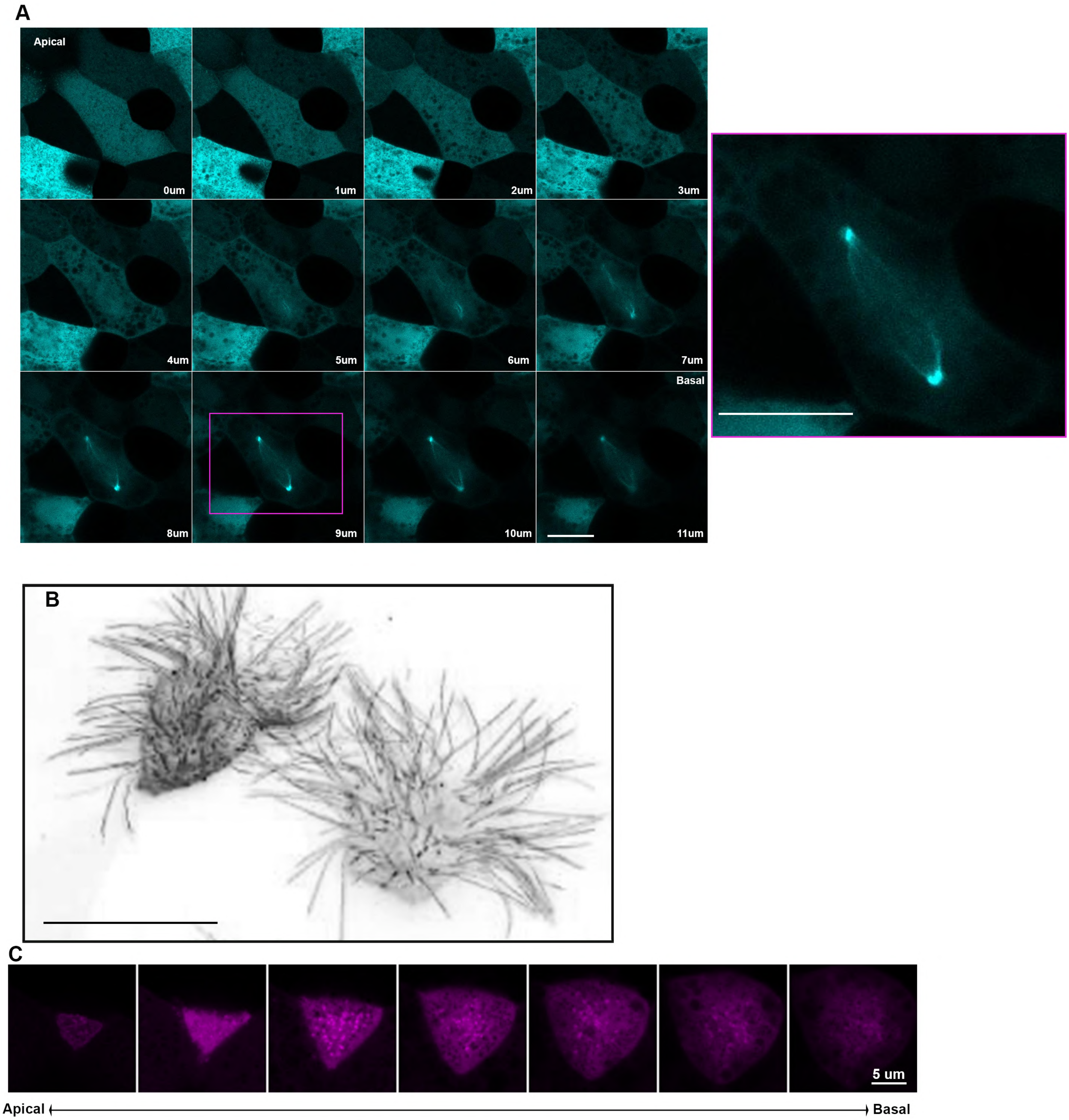

Supplementary Figure 7. Calmodulin localization.
A) Localization of GFP-Calmodulin in a skin epithelial cell undergoing cell division. Zoomed image reveals strong centrosomal localization. B) Localization of GFP-calmodulin in MCCs of stage 32 embryo. Calmodulin displays cilia axonemal localization. C) Localization of GFP-calmodulin in MCCs of stage 18 embryo. Calmodulin displays basal body localization before the docking of basal bodies at the apical cell surface. Scale bars: A,B: 20um; C:5um.

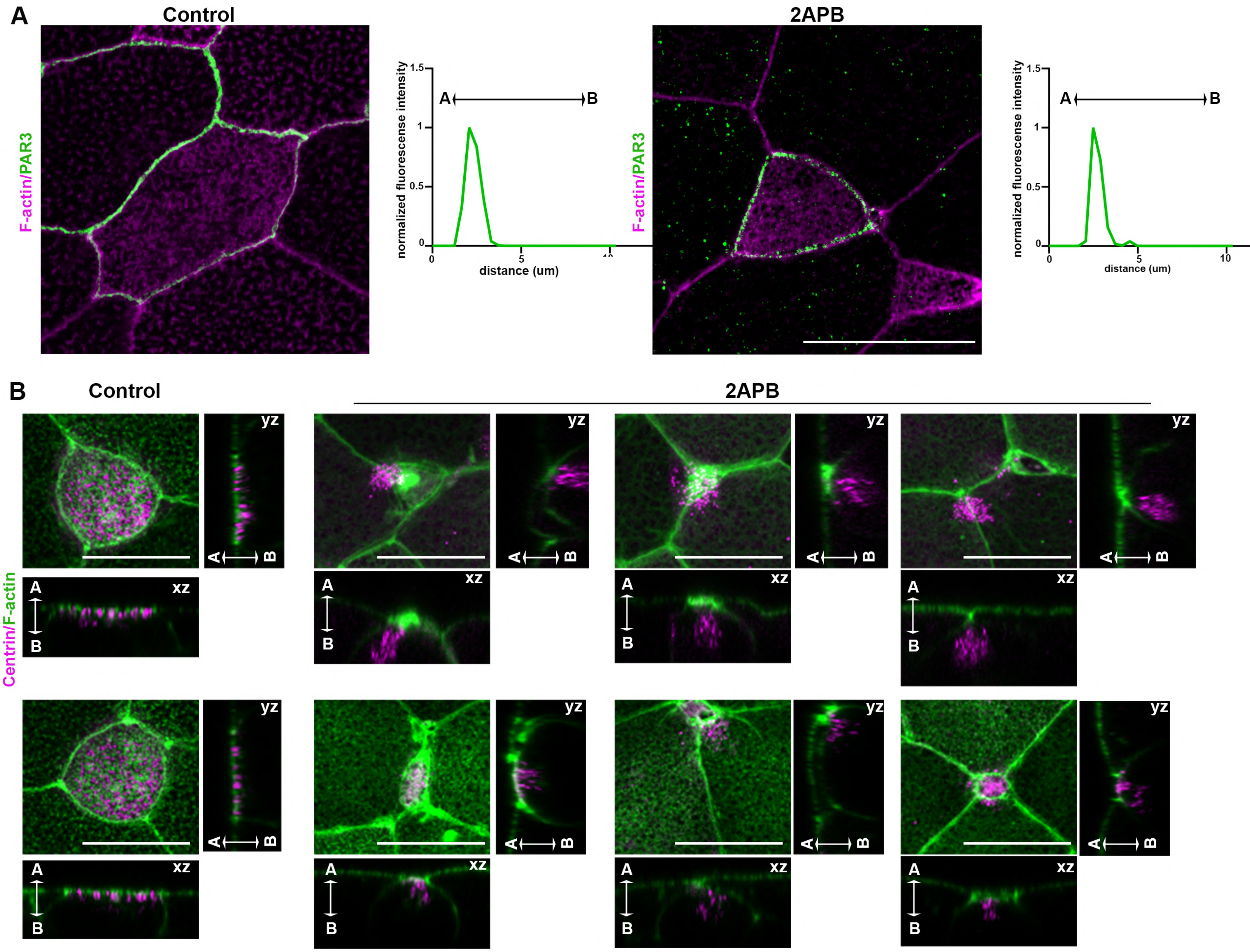

Supplementary Figure 8. Calcium transients are dispensable for apicobasal polarity establishment.
A) Representative images of stage 24 control and 2APB treated embryos expressing Par3-GFP. Fluorescent intensity profiles along the apicobasal axis of MCCs reveal that Par3 apical localization is unaffected by 2APB treatment. B) Representative images of MCCs from stage 23 control and 2APB treated embryos expressing the basal bodies marker centrin. XZ and YZ projections show that even though MCCs in 2APB-treated embryos do not acquire an apical surface, basal bodies in these cells are localized apically. Scale bars: 20um.

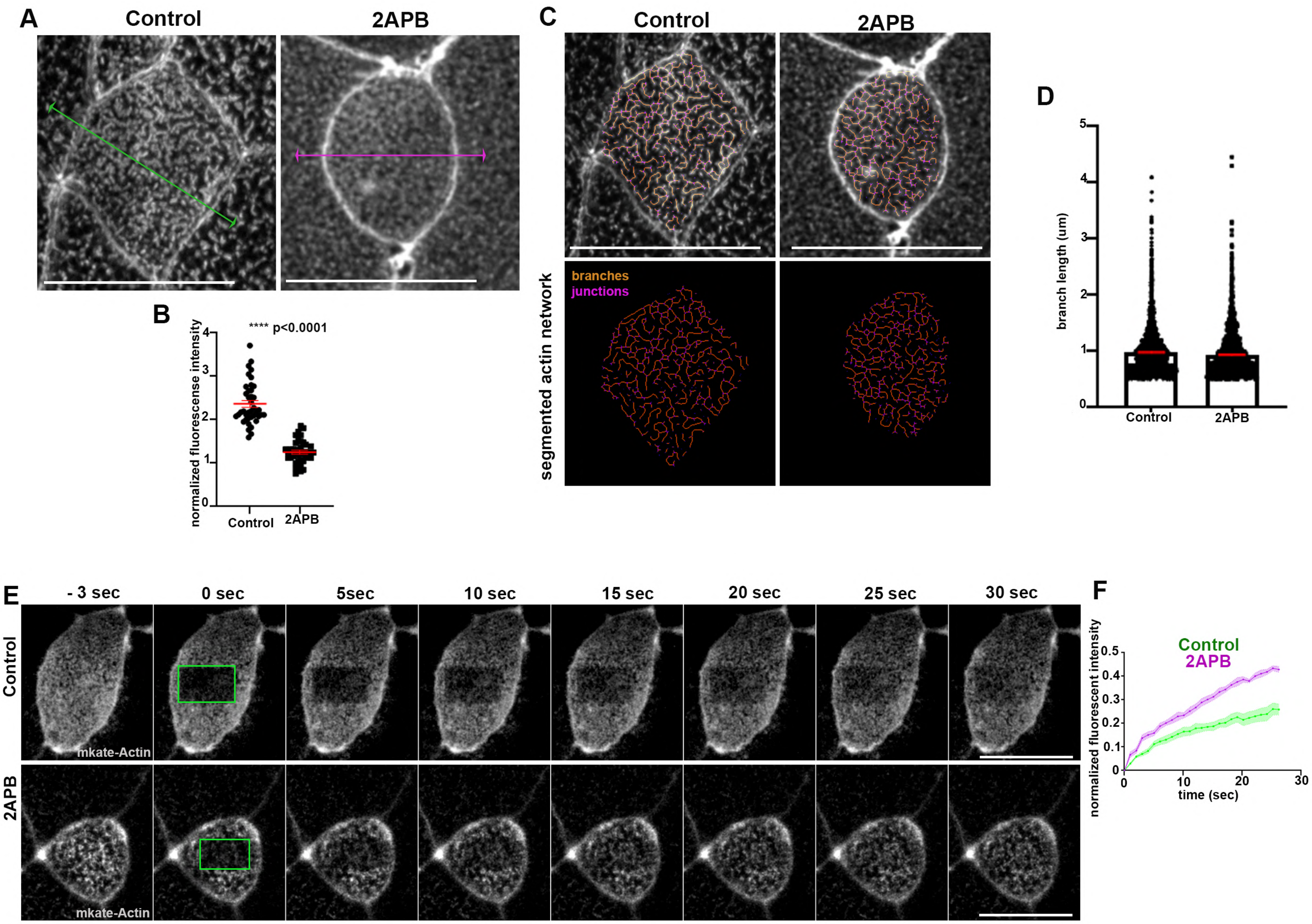

Supplementary Figure 9. Apical actin enrichment but not apical actin network architecture depends on calcium transient generation.

A) Representative images of the apical actin network of MCCs from control and 2APB treated embryos. B) Quantification of apical actin enrichment in MCCs from control and 2APB treated embryos. Two-sided unpaired student's t test; ****$p < 0.0001$; mean ± SEM. n=40 MCCs from 4 control and 40 MCCs from 4 2apb-treated embryos. C) Segmentation of the apical actin network of MCCs reveals that 2APB treatment does not affect the architecture of the apical actin network. Orange: actin filaments. Purple: Actin filament junction points. D) Quantification of the length of actin branches MCCs from control and 2APB treated embryos. Mean for control: 0.984 um; Mean for 2APB:0.9423um. Two-sided unpaired student's t test; **$p = 0.0036$; mean ± SEM. However, Cohen's d= 0.0972, which corresponds to a very small effect size. This suggests that while the difference is statistically significant, the actual difference in actin branch length is minimal. n=2436 branches from control MCCs and 1657 branches from 2APB treated MCCs E) Stills from time lapse recordings from FRAP experiments, showing representative MCCs from control and 2APB treated (12.5um) stage 22 embryo. Green rectangle: Photobleached region. F) Quantification of normalized monomeric actin fluorescent recovery after photobleaching. n=8MCCs from 3 control embryos and 10 MCCs from 3 2apb-treated (12.5um) embryos. Data are presented as mean ± SEM. Scale bars: 20um.
