# Supplementary material for: Calcium transients regulate the apical emergence of basally located progenitors during Xenopus skin development
Source: Nat Commun. 2025 Jul 19;16:6650. doi: 10.1038/s41467-025-61610-7 (PMC12274442; doi:10.1038/s41467-025-61610-7)
Supplement: Supplementary file 3 — Description of Additional Supplementary Files [file 41467_2025_61610_MOESM3_ESM.pdf]

Description of Additional Supplementary Files

File Name: Supplementary Data 1
Description: **Calcium transients in apically emerging MCCs**. Time-lapse recording showing the surface ectoderm epithelium of a control stage 19 embryo. Time interval: 30 sec.

File Name: Supplementary Data 2
Description: **Calcium transients in apically emerging MCCS.** Time-lapse recording showing the surface ectoderm epithelium of a control stage 20 embryo. Time interval: 30 sec.

File Name: Supplementary Data 3
Description: **Calcium transients precede MCC apical surface area expansion.** Time-lapse recording showing an apically emerging MCC from a control stage 19 embryo. Time interval: 30 sec.

File Name: Supplementary Data 4
Description: **Calcium transients are blocked in embryos treated with 2APB.** Time-lapse recording showing the surface ectoderm epithelium of a stage 19 embryo treated with 2APB. Time interval: 30 sec.

File Name: Supplementary Data 5
Description: **2APB treatment blocks MCC epithelial insertion.** Time-lapse recordings of representative MCCs from stage 20 control (left) and 2apb-treated (right) embryos. Time interval: 30 sec.

File Name: Supplementary Data 6
Description: **PACR expression results in defective MCC apical emergence.** 3D reconstruction of a representative stage 24 embryo developed in the dark and expressing PACR.

File Name: Supplementary Data 7
Description: **PLC displays pulsed activity during MCC apical emergence.** Time-lapse recording of a representative MCC from stage 19 embryo. Time interval: 30 sec.

File Name: Supplementary Data 8
Description: **PLC activity is necessary for calcium transient generation.** Time-lapse recordings showing the surface ectoderm epithelium from representative stage 19 control (left) and U73122-treated embryos. Time interval: 30 sec.

File Name: Supplementary Data 9
Description: **Calcium transients precede a transient enrichment of the apical actin network.** Time-lapse recording showing a representative MCC from a stage 21 control embryo. Time interval: 30 sec.

File Name: Supplementary Data 10
Description: **Increase in intracellular calcium leads to enrichment of the apical actin network in MCCs.** Time-lapse recording showing the surface ectoderm epithelium from representative stage 21 control embryos. The recording started after the addition of Thapsigargin. Time interval: 30 sec.

File Name: Supplementary Data 11
Description: **Calcium transients are present during ISC apical emergence.** Time-lapse recording showing the surface ectoderm epithelium from representative stage 24 control embryos. Time interval: 30 sec.

File Name: Supplementary Data 12
Description: FRAP reveals that the actin network is less stable in MCCs from embryos treated with 2APB. Time-lapse recording showing MCCs from control and 2apbtreated stage 22 embryo expressing mKate-actin. Upon photobleaching, fluorescent recovery of mKate-actin is faster in MCCs from embryos treated with 2APB.
