# Supplementary material for: Calcium transients regulate the apical emergence of basally located progenitors during Xenopus skin development
Source: Nat Commun. 2025 Jul 19;16:6650. doi: 10.1038/s41467-025-61610-7 (PMC12274442; doi:10.1038/s41467-025-61610-7)
Supplement: Supplementary file 16 — Reporting Summary [file 41467_2025_61610_MOESM16_ESM.pdf]

nature portfolio

Corresponding author(s): Neophytos Christodoulou
Last updated by author(s): Jun 13, 2025

Reporting Summary

Nature Portfolio wishes to improve the reproducibility of the work that we publish. This form provides structure for consistency and transparency in reporting. For further information on Nature Portfolio policies, see our Editorial Policies and the Editorial Policy Checklist.

Statistics

For all statistical analyses, confirm that the following items are present in the figure legend, table legend, main text, or Methods section.

n/a	Confirmed	
☐	☒	The exact sample size (*n*) for each experimental group/condition, given as a discrete number and unit of measurement
☐	☒	A statement on whether measurements were taken from distinct samples or whether the same sample was measured repeatedly
☐	☒	The statistical test(s) used AND whether they are one- or two-sided<br>*Only common tests should be described solely by name; describe more complex techniques in the Methods section.*
☒	☐	A description of all covariates tested
☒	☐	A description of any assumptions or corrections, such as tests of normality and adjustment for multiple comparisons
☐	☒	A full description of the statistical parameters including central tendency (e.g. means) or other basic estimates (e.g. regression coefficient) AND variation (e.g. standard deviation) or associated estimates of uncertainty (e.g. confidence intervals)
☒	☐	For null hypothesis testing, the test statistic (e.g. *F*, *t*, *r*) with confidence intervals, effect sizes, degrees of freedom and *P* value noted<br>*Give P values as exact values whenever suitable.*
☒	☐	For Bayesian analysis, information on the choice of priors and Markov chain Monte Carlo settings
☒	☐	For hierarchical and complex designs, identification of the appropriate level for tests and full reporting of outcomes
☒	☐	Estimates of effect sizes (e.g. Cohen's *d*, Pearson's *r*), indicating how they were calculated

Our web collection on statistics for biologists contains articles on many of the points above.

Software and code

Policy information about availability of computer code

Data collection	ZEISS LSM 710
Data analysis	FIJI software, ZEN (black edition), GraphPad Prism 8.0

For manuscripts utilizing custom algorithms or software that are central to the research but not yet described in published literature, software must be made available to editors and reviewers. We strongly encourage code deposition in a community repository (e.g. GitHub). See the Nature Portfolio guidelines for submitting code & software for further information.

Data

Policy information about availability of data

All manuscripts must include a data availability statement. This statement should provide the following information, where applicable:

- Accession codes, unique identifiers, or web links for publicly available datasets
- A description of any restrictions on data availability
- For clinical datasets or third party data, please ensure that the statement adheres to our policy

The authors declare that all data supporting the findings of this study are available within the article and its supplementary information files or from the corresponding author upon reasonable request.

Research involving human participants, their data, or biological material

Policy information about studies with human participants or human data. See also policy information about sex, gender (identity/presentation), and sexual orientation and race, ethnicity and racism.

Reporting on sex and gender	N/A
Reporting on race, ethnicity, or other socially relevant groupings	N/A
Population characteristics	N/A
Recruitment	N/A
Ethics oversight	N/A

Note that full information on the approval of the study protocol must also be provided in the manuscript.

Field-specific reporting

Please select the one below that is the best fit for your research. If you are not sure, read the appropriate sections before making your selection.

[x] Life sciences [ ] Behavioural & social sciences [ ] Ecological, evolutionary & environmental sciences

For a reference copy of the document with all sections, see nature.com/documents/nr-reporting-summary-flat.pdf

Life sciences study design

All studies must disclose on these points even when the disclosure is negative.

Sample size	No sample-size calculation was performed. Sample size was determined based on our previous experience and the work of other groups using frog embryos as an experimental model system.
Data exclusions	No data were excluded from the analysis.
Replication	In the study, all attempts at replication were successful.<br>Each result described in this paper is based on at least two independent biological replicates. Most of the data presented are based on but on more than two independent experiments.
Randomization	Samples (frog embryos) were allocated randomly into experimental groups
Blinding	Blinding is not relevant to this study.

Reporting for specific materials, systems and methods

We require information from authors about some types of materials, experimental systems and methods used in many studies. Here, indicate whether each material, system or method listed is relevant to your study. If you are not sure if a list item applies to your research, read the appropriate section before selecting a response.

Materials & experimental systems

n/a	Involved in the study	
[ ]	[x]	Antibodies
[x]	[ ]	Eukaryotic cell lines
[x]	[ ]	Palaeontology and archaeology
[ ]	[x]	Animals and other organisms
[x]	[ ]	Clinical data
[x]	[ ]	Dual use research of concern
[x]	[ ]	Plants

Methods

n/a	Involved in the study	
[x]	[ ]	ChIP-seq
[x]	[ ]	Flow cytometry
[x]	[ ]	MRI-based neuroimaging

Antibodies

Antibodies used	Primary antibodies used:<br>acetylated tubulin (sc-23950, Santa Cruz Biotechhnology)<br>β-catenin (11279-R021, SinoBiological)

	E-cadherin (5D3, DSHB)<br>ZO-1 (21773-1-AP, Proteintech).
Validation	The subcellular localization of all the proteins analyzed in this study has been previously reported. This was used to validate the specificity of the antibody.<br>Primary Antibodies used in whole mount immunofluorescence:<br>ZO-1: displayed tight junction localisation as described before (doi: 10.1083/jcb.202303052).<br>E-cadherin: it correctly stained adherens junction (doi:10.1016/j.ydbio.2019.03.010).<br>β-catenin: displayed adherens junction localization (https://www.sinobiological.com/antibodies/human-beta-catenin-11279-r021)<br>acetylated tubulin: stained the ciliary axoneme as previously reported (doi: 10.1083/jcb.202303052).

Animals and other research organisms

Policy information about studies involving animals; ARRIVE guidelines recommended for reporting animal research, and Sex and Gender in Research

Laboratory animals	Adult Xenopus laevis animals for collection of embryos.
Wild animals	N/A
Reporting on sex	N/A
Field-collected samples	N/A
Ethics oversight	The procedures used for derivation of Xenopus embryos from adult animals were approved by the National Committee for the Protection of Animals Used for Scientific Purposes of the Republic of Cyprus with the license (CY/EXP/PR.L05/2022)

Note that full information on the approval of the study protocol must also be provided in the manuscript.

Plants

Seed stocks	N/A
Novel plant genotypes	N/A
Authentication	N/A
